# Menstrual Blood Stem Cell Transplantation in Mice Model of Acute Liver Failure: Does Gender of Recipient Affect the Outcome?

**Published:** 2019

**Authors:** Mina Fathi-Kazerooni, Gholamreza Tavoosidana

**Affiliations:** Department of Molecular Medicine, Faculty of Advanced Technologies in Medicine, Tehran University of Medical Sciences, Tehran, Iran

**Keywords:** Gender, Failure, Regenerative medicine, Stem cells

## Abstract

**Background:**

There exists a dramatic rise in liver failure and numerous patients undergo liver transplant for life-saving reasons annually. Introducing alternatives to allo-graft transplantation is necessary due to present limitations. Recently, a noninvasive stem cell population from Menstrual blood-derived Stem Cells (MenSCs) has been identified. There is an increasing interest in the application of MenSCs in tissue engineering; however, the fact that these gender-specific stem cells are safe for use in male sex is still not well defined.

**Methods:**

In this research, a model of acute liver failure was created in male and female immunocompetent Balb-C mice through intraperitoneal injection of Carbon tetrachlo-ride (CCl_4_ ) and MenSCs were transplanted intravenously 48 *hrs* after induction of liver injury to evaluate their therapeutic potential. All mice were sacrificed on days 1, 7, and 30 post-transplantation to examine biochemical and molecular markers and pathological appearances.

**Results:**

Results showed the liver engraftment of MenSCs by immunofluorescence staining using anti-human mitochondrial antibody in both male and female treated groups. The restoration of serum markers of liver injury, aspartate aminotransferase and ala-nine aminotransferase, as well as expression levels of liver-specific genes, tyrosine aminotransferase and cholesterol 7 alpha-hydroxylase, were more significant in the female treated group compared with the male treated group on day 7 (p<0.05); however, after 30 days, there were no significant differences. Furthermore, hematoxylin and eosin and periodic acid-Schiff staining of liver sections demonstrated the considerable liver regeneration post cell therapy in both groups. Notably, data has shown that MenSCs could engraft into injured liver tissues and result in the same effect in the regeneration of liver function in both genders.

**Conclusion:**

Results of this study introduce MenSCs therapy as an attractive alternative approach for liver repairing and regeneration which has no gender constraints.

## Introduction

The liver, the largest body gland, is exposed to various risk factors including alcoholism, obesity, diabetes, toxins, autoimmune diseases and hereditary conditions 1. Acute Hepatic Failure (AHF) is a clinical syndrome with a high mortality rate 2. Liver transplantation is the most common therapy but its application is limited for lack of available donors, high costs and risks of organ rejection and lifelong immunosuppressive medications 3. Epidemiological studies have introduced male gender as an independent predictor of liver fibrosis progression towards cirrhosis in hepatitis B and C-virus, as well as non-alcoholic steatohepatitis 4. In addition, studies showed that physiopathology of liver disease is different in male and female patients and several potential mechanisms have not been identified yet.

In recent years, cell therapy with mesenchymal stem cells is considered an alternative treatment to allograft transplantation 5. Till now, many studies have been conducted on the use of mesenchymal stem cells derived from human bone marrow, adipose tissue and umbilical cord blood in liver regeneration 6–8. Nevertheless, problems such as limited availability, invasive sample collection, and low proliferation capacity limit the applicability of these adult stem cells 9.

In recent years, a noninvasive stem cell population from Menstrual blood-derived Stem Cells (MenSCs) with several advantages including high accessibility, renewability, sustainability, and low production costs which can be used without ethical concerns, has been identified 10,11. This type of stem cells expressed some markers of both mesenchymal and embryonic stem cells and could differentiate into three germ layers 10,12,13.

Recently, several studies reported that gender-specific differences in mesenchymal stem cells from different sources could impact the potential of proliferation and differentiation of cells 14,15. Furthermore, recent studies have shown that the secretome of mesenchymal stem cells isolated from different gender and sources may present significant variations 14. These differences in stem cell potential may influence recipient responses and outcome of cell transplantation 16. Additionally, large data about transplant failure in hematologic stem cell transplantation resulted in HLA mismatch between donor and recipient 17–19. However, some studies reported that transplantation of immature stem cells such as umbilical cord stem cells could be tolerated by the recipient despite an HLA mismatch 20.

There are *in vitro* and *in vivo* studies which report the potential of differentiation of MenSCs into hepatocyte-like cells and restoration of AHF with transplantation of these stem cells in mice model 11,21, but there are no data about the outcome of MenSCs transplantation into a male recipient. The most remarkable aspect about the MenSCs is that they are gender-specific cells with several general variations with male cells which must be acknowledged prior to transplantation of them into the male recipients. In addition, a greater understanding of the sex effect on diverse stem cell populations is required to improve their ultimate clinical efficacy. Therefore, in this study, an attempt was made to compare the effectiveness of MenSCs transplantation on the restoration of AHF in male and female immunocompetent Balb/C mice with acute liver failure. The recipient's gender was considered as an important factor in predicting the effect of MenSCs transplantation on reducing the effects of toxic liver agents. The therapeutic effect of MenSCs was assessed by evaluation of the biochemical, histopathological, and molecular hepatic factors in both genders at different times.

## Materials and Methods

### Isolation and culture of MenSCs

Donors for menstrual blood were selected from healthy females aged 20–35 years. All donors signed an informed consent approved by the medical ethics committee of Avicenna Research Institute. About 5 *ml* of menstrual blood was collected using a Diva cup (Di-va International Co., Lunette, Finland) during the first 2 days of the menstrual cycle. The contents of Diva cup were collected in a collection tube containing 2.5 *mg/ml* fungizone (Gibco, UK), 100 *mg/ml* streptomycin, 100 *U/ml* penicillin (Sigma-Aldrich, St. Louis, MO, USA), and 0.5 *mM* EDTA in Phosphate Buffered Saline (PBS).

MenSCs were isolated and cultured from menstrual blood by the method described previously 22. Briefly, menstrual blood mononuclear cells were separated by Ficoll-Hypaque (GE-Healthcare, Uppsala, Sweden) density gradient. After washing with PBS, the cell pellet was subsequently cultured in a T75 flask containing complete Dulbecco’s modified Eagle’s medium-F12 (DMEM-F12; Sigma-Aldrich) supplemented with 10% FBS, 2 *mM* L-glutamine, 100×NEAA, 100 *U/ml* penicillin, 100 *mg/ml* streptomycin, and 25 *mg/ml* fungizone and maintained at 37°*C* in a 5% humidified CO_2_ incubator for approximately 2 days. Following initial incubation, the non-adherent cells were washed away, leaving behind the adherent cell population. 4–7 days later, the first colonies appeared. After achieving 70–80% confluence, adherent cells were passaged using Trypsin/EDTA (Gibco).

### Immunophenotyping of MenSCs

The cultured MenSCs were detected via the expression of mesenchymal stem cell markers such as CD73, CD105, CD146, CD10, CD29, hematopoietic stem cell markers such CD34 and CD133, and embryonic stem cell marker including OCT4, SSEA-4 using a flow cytometric analysis, as described in our previous paper 13. About 10^5^ cells/100 *μl* were separately incubated with PE-conjugated monoclonal mouse anti-human CD73 (BD Pharmingen, CA, USA), CD105 (BioLe-gend, CA, USA), CD146 (clone P1H12; BD Pharmin-gen, USA), CD10 (BioLegend, CA, USA), CD29 (BioLegend, CA, USA), CD34 (BD Pharmingen, USA), CD133 (BD Pharmingen, USA), and SSEA-4 (eBioscience, CA, USA) for 40 *min* at 4°*C*. For OCT-4, permeabilization of the cell membrane was done by 0.1% saponin. Cells were then incubated with the anti-human OCT-4 antibody (Abcam, CA, USA) for 40 *min*. Finally, all cells were fixed in 1% formaldehyde solution and marker expressions were evaluated using flow cytometry (Partec, Germany) with reference to appropriate isotype controls.

### *In vitro* differentiation of MenSCs

MenSCs (Passage 3–4) were plated in 24-well plates with DMEM supplemented with 15% Fetal Bovine Serum (FBS) and used for the differentiation study when reached 70–80% confluence. Osteogenic, chondrogenic, and adipogenic differentiation was induced by the differentiation media as described previously 11,22. Correct differentiations were confirmed by Alizarin red staining (Sigma-Aldrich) for mineralized calcium, immunohisto-chemistry using primary monoclonal mouse anti-human Collagen type II (Clone 5B2.5, 1:500; Ab-cam) and secondary antibody FITC-labeled goat anti-mouse IgG (Abcam), and oil red O staining for lipid droplet according to previously described protocols 11,22.

### Animal model of acute liver injury and MenSCs transplantation

To induce acute liver failure, 1 *ml/kg* body weight Carbon tetrachloride (CCl_4_) mixed with mineral oil (1:10 ratio) was injected intraperitoneally into 8–10 week old male and female Balb/C mice which were maintained in the animal center at the Avicenna Research Institute according to animal care guidelines. After 48 *hr*, all CCl_4_ treated mice were randomized and allocated to three groups: acute liver injury model group as the control group (male gender n=5, female gender n=5), male MenSCs-transplanted group (n=15), and female MenSCs-transplanted group (n=15).

Subsequently, all mice of the acute liver injury model group received a transfusion of 0.2 *ml* PBS, whereas MenSCs transplantation groups received a transfusion of 8×10^5^ stem cells suspended in 0.2 *ml* of PBS through tail vein injection, respectively. 5 mice in each gender without injection of CCl_4_ were regarded as the normal group.

At 1, 7, and 30 days following cell transplantation, blood samples, and liver tissue were collected from mice in all treated groups for further analysis. The fresh liver tissues were collected for further studies such as Hematoxylin and Eosin (HE) and immunohis-tochemical staining and Reverse Transcription Polyme-rase Chain Reaction (RT-PCR).

### Liver function assay

Blood samples were centrifuged and the serum was collected after 1, 7 and 30 days following cells transplantation. The levels of Alanine aminotransferase (ALT), Aspartate aminotransferase (AST), total bilirubin, albumin, and urea in the serum were measured using an automatic biochemical analyzer (Roche Diagnostic GmbH, Mannheim, Bad Nauheim, Germany).

### Histopathological analysis

Liver lobes were collected from mice at each of the following 3 time points of 1, 7, and 30 days after the injection of MenSCs. The liver tissues were fixed overnight in 10% formalin, dehydrated in graded ethanol, embedded in paraffin (Merck, Germany), cut into serial 5 *μm* sections, and HE stained (Sigma-Aldrich).

### Evaluation of glycogen storage ability

Paraffin-embedded liver lobes were cut to 4–5 *μm* sections, deparaffinized, hydrated to water, and oxidized in 0.5% periodic acid solution for 5 *min*. Then, the slides were placed in Schiff reagent for 15 *min* and after washing, counterstained in Mayer’s hematoxylin for 1 *min*. Slides were then dehydrated and cover-slipped using a synthetic mounting medium and observed under a phase-contrast microscope (Olympus BX51). The intra-cellular glycogen was stained purple and the nuclei blue.

### Real-time quantitative polymerase chain reaction

At the time of sacrifice, part of each fresh liver sample was frozen immediately in liquid nitrogen to preserve good quality RNA for gene expression studies. Then the samples were transferred by nitrogen tank from the surgery room and kept at −80°*C* until use.

Total RNA was extracted from the liver sections using AccuZol reagent according to manufacturer’s instruction. First-strand cDNA was synthesized by using 1 *μg* DNAse-treated RNA, 1 *μL* Super ScriptTM II Reverse Transcriptase (200 *U*), 20 *pM* N6 Random-Hexamer, 20 *pM* dNTP Mix, 4 *μL* 5X First-Strand buffer, 2 *μL* Dithiothreitol (0.1 *M*), and 1 *μL* RiboLock TMRNase inhibitor in a thermocycler (Eppendorf, Germany) at 25°*C* for 10 *min*, 42°*C* for 50 *min*, and 70°*C* for 15 *min*. Next, qRT-PCR was performed using ABI 7500 real-time PCR as follows: initial denaturation at 95°*C* for 10 *s*, 40 cycles of a two-step PCR (95°*C* for 5 *s*, 60°*C* for 30 *s*), dissociation stage at 95 °*C* for 15 *s*, 60°*C* for 1 *min* and 95°*C* for 15 *s*. The primers used are listed in table 1.

**Table 1.** Sequences of the primers used in the qRT-PCR analysis

**Gene of interest**	**Sequence**	**Product size (*bp*)**	**NCBI Accession No.**
***TAT***	F:5′ -CGCTTCCTATTACCACTGTCC - 3′	167	NM_146214
	R:5′- ACTCAGCCAATGTCCTGTAGA-3′		
***CK-18***	F:5′-GTGAAGAGCCTGGAAACTGAGA -3′	283	NM_010664
	R: 5′-CATCTACCACCTTGCGGAGT-3′		
***ALB***	F:5′-AAGGCTACAGCGGAGCAAC-3′	117	NM_009654
	R:5′-GACAAGGTTTGGACCCTCAGTC -3′		
***CYP7A1***	F:5′-ACAACGGGTTGATTCCATACC -3′	228	NM_007824
	R: 5′-GTCCAAATGCCTTCGCAGA -3′		
***B-Actin***	F: 5′-GTCGAGTCGCGTCCACC -3′	114	NM_007393
	R: 5′- CATTCCCACCATCACACCCTG -3′		

### Tracking of MenSCs homing by immunostaining

The capability of transplanted stem cells for migration and homing in injured liver tissue was assessed by immunohistochemistry. The sections were deparafinized and rinsed with Tris-Buffered Saline (TBS) and blocked with 3% H_2_O_2_. After blocking the endogenous biotin with Biotin-Blocking System (Cat number: X0-590; Dako), the slides were blocked in mouse serum for 45 *min* at 4°*C* and then were incubated overnight at 4°*C* with the mouse anti-human anti-mitochondrial antibody (MAB1273B, 1:150; Millipore). As a negative control, sections were stained with isotype control antibody (IgG1).

Finally, the slides were rinsed with TBS and 1% Bovine Serum Albumin (BSA) and incubated with horse-radish peroxidase (HRP)-Streptavidin Conjugate (Cat number: 43-4323, 1:150; Invitrogen) at RT for 1 *hr*. Positive transplanted MenSCs were detected using HRPDAB Detection System (Cat number: ab64264; Ab cam). After counter-staining with Mayer’s hematoxylin, the slides were monitored under the phase-contrast microscope.

### Statistical analysis

The data were presented as mean±Standard Deviation (SD). The quantitative results were analyzed by SPSS 13 statistical software. Multiple comparisons among means were performed using

ANOVA and student’s t-test was used for comparing the means of two groups. Mean efficiencies and crossing point values for each gene were determined with LinRegPCR (Version 11.0) using relative expression software tool-2009 (REST-2009). A p-value<0.05 denoted a statistically significant difference.

## Results

### In vitro characterization of isolated MenSCs

**Figure 1.**
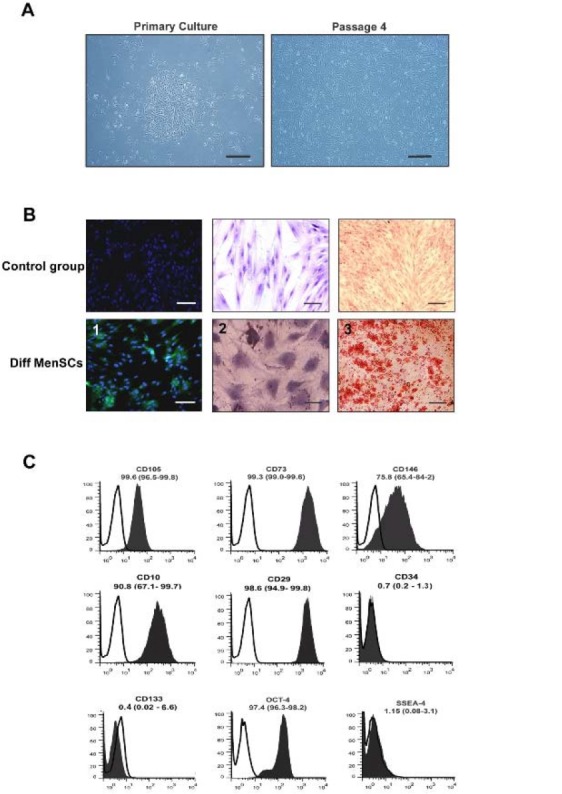
A. Characterization of cell morphology in MenSCs in different passages: The isolated cells exhibited spindle-shape morphology. Scale bar: 100 μm B: MenSC differentiation into chondrocytes (1), adipocytes (2), and osteoblast (3) judged by immunostaining of collagen type II and oil red O staining, and alizarin red staining, respectively. Scale bar=100 *mm*. C: Characterization of CD markers in MenSCs (black) and in isotype controls (white). Data represent the means ± SD (n=5).

Adherent mononuclear cell, isolated from menstrual blood exhibited spindle-shaped or fibroblastic morphology as shown in figure 1A. These cells reached 70–80% confluency during 10±3 days. The analysis of flow cytometry result showed that 99.6, 99.3, 75.8, 90.8, and 98.6% of the cells were CD105, CD73, CD146, CD10, and CD29 positive (Mesenchymal stem cell markers), respectively. The immunophenotyping also revealed high expression of OCT-4 and the lack of the surface marker SSEA-4, CD34, and CD133 (Figure 1C).

As shown in figure 1C, MenSCs were differentiated into mesodermal lineages as shown by collagen II-expressing chondrocyte-like cells and mineralized calcium producing osteoblasts. However, MenSCs show-ed a low potential for differentiation into adipocytes (Figure 1B).

### Establishment of acute liver failure

Based on the result of previous study 21, one dose of 1 *ml/kg* body weight CCl4 10% in mineral oil was injected intraperitoneally into 8–10 week old Balb/C mice. After 48 *hr*, the mice showed less activity, energy, and aggressiveness. Necropsy of mice with CCl_4_ injection showed severe adhesion in the peritoneal cavity associated with ascites. The liver tissue surface was inflamed and coarse in texture with multiple nodules and a pale discoloration.

**Figure 2.**
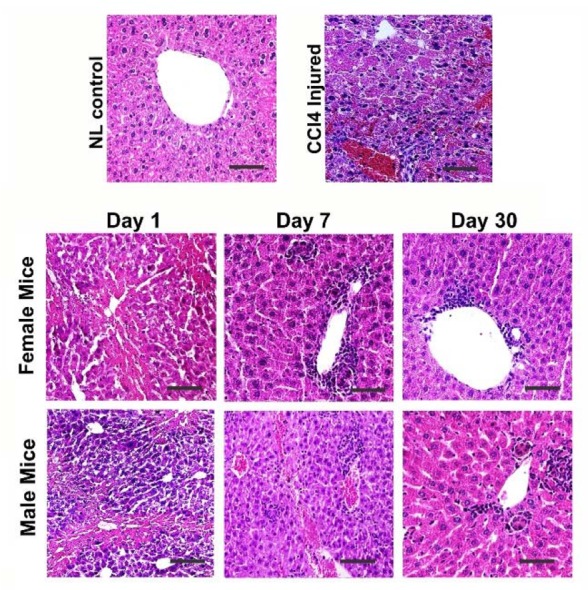
Comparison of liver histology between the control, liver failure model, and male and female cell-treated groups. CCl4 treated group showed hepatocyte cytoplasm vacuolization, and infiltration of inflammatory cells. During 30 days the cell transplantation significantly ameliorated liver injury without any significant difference between both genders. Scale bar: 100 *μm*.

HE staining of CCl_4_-treated liver tissues showed the multifocal areas of coagulation necrosis that occurred primarily in centrilobular zones with some foci of necrosis diffusely dispersed to mid-lobular and periportal areas. However, in zone I and II, the most prominent features were vacuolar change and hydropic degeneration of hepatocytes. Infiltration of inflammatory cells and activation of Kupffer cells were seen in areas of hepatic injury. Some foci of hemorrhage were also noted (Figure 2).

Liver function assay of mice with liver failure showed that serum levels of liver enzymes including AST, ALT, total bilirubin, and urea collected 48 *hr* after injection were significantly (p<0.05) elevated compared to the control healthy group (Table 2). All the above data indicated that AHF occurred.

**Table 2.** Liver enzymes analysis in healthy control and liver failure groups

	AST (U/L)^*^	ALT (U/L)^*^	Total Bilirubin^*^	Urea^*^	**Albumin**
**Normal control group**	98.66±15.63	37.66±5.20	0.41±0.13	45.76±5.14	3.16±1.30
**Liver failure control group**	975.00±25.00	1149.50±12.41	0.78±0.24	67.36±4.23	3.62±2.28

48 *hr* after CCl_4_ injection the analysis of serum was performed. 5 mice from each group were analyzed;

* p<0.05.

### MenSCs transplantation improved the function of CCl_4_ injured liver in both genders

Based on the data of the previous study 21, the mice underwent intravenous tail vein transplantation of 8 ×10^5^ MenSCs, 48 *hr* following CCl4 induced injury, while the control group received an injection of PBS. The mice were sacrificed on days 1, 7 and 30 after cells transplantation.

**Figure 3.**
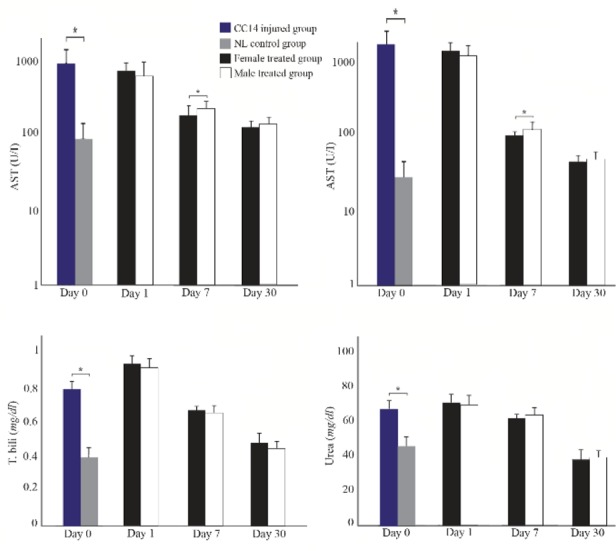
Liver function analysis in male and female mice after MenSCs transplantations in different point times. Data were expressed as the means ± SD. ^*^p< 0.05.

As shown in figure 3, in both cell treated groups, the levels of serum markers including AST, ALT, total bilirubin, and urea revealed a significant gradual decreasing trend over the treatment time. Statistically, there were no significant differences in the levels of these markers among the cell treated groups and CCl_4_ injured group 1 day following cell transplantation. However, 7 and 30 days following transplantation, the levels of AST, ALT, total bilirubin, and urea in the male and female MenSCs-treated groups were significantly lower compared with those in the control CCl_4_ treated group (p<0.05). Notably, the female MenSCs-treated group showed lower levels of AST and ALT compared with those in the male MenSCs-treated group (p<0.05) 7 days after transplantation, but there was no significant difference between the treated groups in 30 days. The serum levels of total bilirubin, and urea in both female and male transplanted mice had the same reduction after 7 and 30 days (p>0.05). In 30 days following transplantation, the levels of these serum markers decreased to their lowest level (p<0.05). Furthermore, the level of albumin in the CCl_4_ treated group was slightly higher than that in both cell treated groups at three time points; however, the differences between the groups were not statistically significant.

These data suggested that MenSCs transplantation could significantly decrease the serum level of liver injury markers, which indicate their efficiency in liver regeneration in both genders.

### MenSCs transplantation alleviates the histopathological features of liver damage in both genders

Microscopical examination of liver on day 1 after transplantation showed expansive vacuolar degeneration, infiltration of inflammatory cell in the periportal area, and necrosis. There was no noticeable difference between the two cells treated groups (Figure 2). On day 7 following transplantation, tissue damage and necrosis happened to a lesser extent in both cell treated groups compared to CCl_4_ group. Additionally, in both groups, minor derangement of the hepatocyte cord and portal hepatitis was noted compared to CCl_4_ treated group. Slight bile duct hyperplasia, vascular congestion with some foci of necrotic cells and hemorrhage were also observed. At this time point, no significant difference was observed in the histopathology of male and female MenSCs-transplanted groups (Figure 2). However, after 30 days post-transplantation, there was a significant improvement in the histopathological appearance of the liver. Mild hepatocyte vacuolation, minimal infiltration of inflammatory cells and mild to moderate congestion were seen in the male treated group (Figure 2). While microscopical examination of the female transplanted group exhibited clear hepatic recovery characterized by a relatively satisfactory regeneration of hepatocytes, few cells were damaged (Figure 2).

### Recovery of glycogen storage ability in MenSCs-treated liver tissues

**Figure 4.**
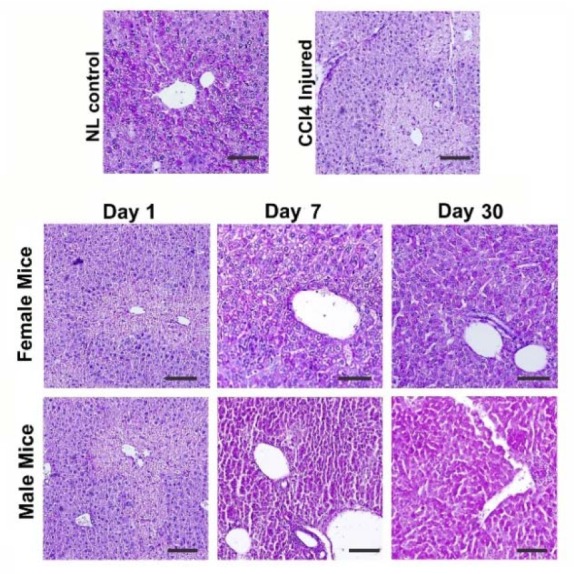
Comparison of liver tissue glycogen content between male and female mice treated with MenSCs. There was no notable difference between the two genders.

Liver tissues from mice with AHF showed notable depletion of glycogen deposition and coagulative necrosis (Figure 4). After 1 day, there was no significant recovery in both MenSCs-treated groups; however, on day 7, male and female treated groups showed a partial increase in the intensity of PAS positivity especially in the periportal areas (Figure 4).

After 1 month, a moderate PAS positivity was observed in hepatic lobules of the female MenSCs-treated group; and male MenSCs-treated group demonstrated a noticeable improvement in glycogen content (Figure 4). These data from microscopical examination of PAS-stained liver sections demonstrated that MenSCs transplantation could recover glycogen storage ability of CCl_4_-injured livers in both genders without significant difference.

### Expression levels of hepatic genes improved following MenSCs transplantation in both genders

**Figure 5.**
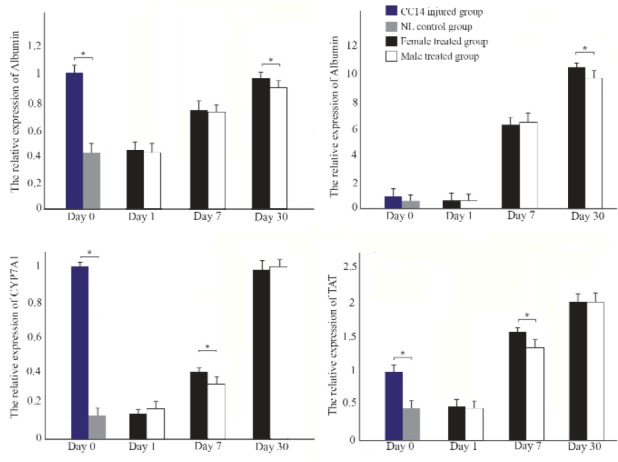
RT-PCR detection of the mRNA expression of hepatic genes in liver tissues from different groups. Data were normalized to corresponding β-Actin and calculated in reference to control normal (NL) group. ^*^p<0.05.

Real-time quantitative PCR analysis was performed to detect the expression levels of albumin, CK-18, cyto-chrome P450 family 7 subfamily A member 1 (CYP-7A1), and TAT in mice liver tissues. In CCl_4_ control group, the expression level of albumin decreased significantly while in both cell treated groups the level of this liver-specific marker gradually increased from day 1 to 30 following transplantation and the female MenSCs-treated group showed the highest level of albumin after 30 days of treatment (p<0.05) (Figure 5).

Similarly, CK-18 mRNA showed marked down-re-gulation following CCl_4_ injection and gradually up-regulated from days 1 to 30 post cell transplantation. In 30 days following transplantation, the expression of CK-18 exhibited significant up-regulation in the female MenSCs-treated group compared to the male MenSCs-treated group (p<0.05). In addition, the expression of CYP7A1 and TAT genes at the mRNA level showed significant up-regulation in 1 month following cell transplantation in both cells treated groups (p<0.05). Also, comparison between both cells treated groups showed that the female MenSCs-transplanted group had significantly greater expression of CYP7A1 and TAT compared with the male MenSCs-treated group on day 7 post-transplantation (p<0.05); however, there was no significant difference in TAT and CYP7A1 gene expression level between two treated groups on day 30 (Figure 5).

### Localization of transplanted MenSCs in injured liver

**Figure 6.**
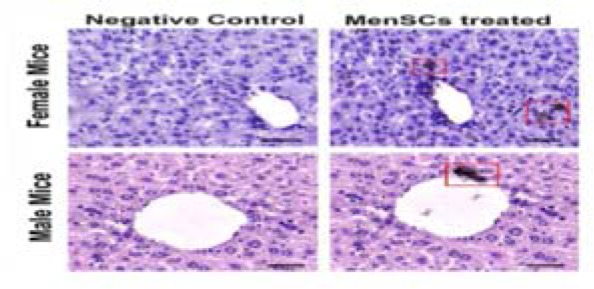
Immunohistochemical staining of mitochondrial protein (red box) in liver samples retrieved from male and female MenSCs-treated groups after 7 days. Liver section from normal mice served as negative control. Scale bar: 100 *μm*.

To localize the infused MenSCs in the CCl_4_-injured liver, liver tissues were immunohistochemically stained using anti-human mitochondrial antibody. It was observed that 7 days after transplantation, MenSCs could engraft into the portal areas of the injured liver in both genders, especially around portal areas (Figure 6).

## Discussion

In the last decade, various types of stem cells have been introduced and their differentiation into three germ layers cells has been investigated. Mesenchymal stem cell therapy, with the characteristics of anti-inflammation, immunomodulation and trophic effects, has been proposed as an alternative treatment for orthotropic liver transplantation 23,24.

In the present study, MSCs were successfully isolated from menstrual blood samples, obtained from healthy women during the first days of menstrual cycles. The experimental acute liver injury was induced by intraperitoneal CCl_4_ injection. Consistent with previous studies, no deaths were observed in CCl_4_ treated group after transplantation of MenSCs in both male and female immunocompetent Balb/C mice, while without cell therapy all mice died during the first 48 *hr* 25,26. The efficacy of MenSCs transplantation was investigated by improvement in liver biochemical functions, the decrease in hepatocytes degradation, and alleviating the histo-pathological liver tissue damage in both male and female groups.

It has been demonstrated that MSCs could promote liver tissue regeneration through inhibition of inflammation, reduction of apoptosis, suppressing T lymphocytes and intrahepatic Natural Killer cells (NKs), and modulation of Hepatic Stellate Cells (HSCs) 5,27. Previous studies have suggested two possible mechanisms for stem cell therapy for repairing damage to the liver; migration, engraftment, and transdifferentiation of stem cells at the site of cell injury or stem cells fusion with host hepatocytes, and the paracrine secretion of various cytokines and growth factors such as HGF, Vascular Endothelial Growth factor (VEGF), interleukin-6, and insulin-like growth factor binding proteins that stimulate revascularization and endogenous cell proliferation 28,29.

Here, engraftment of transplanted MenSCs into injured areas of the liver host of both genders were shown; however, due to the rapid improvement in the liver tissue regeneration in a short time (1 week), MenSCs homing and transdifferentiation seem unlikely to be the main mechanism of liver tissue repair after cell transplantation. Furthermore, previous studies manifested that about 1 to 3% of the host liver were repopulated by donor stem cells and about 4 to 5% of transplanted stem cells were engrafted into the recipient's liver during 1 month following transplantation 5. Therefore, it can be concluded that the trophic effect of transplanted stem cells may be the main mechanism of hepatic tissue regeneration.

Recent studies revealed that sex differences are an important factor that affects the properties of some adult stem cells. Crisostomo *et al* demonstrated that there are differences in the activation of the MSCs in the two sexes. They stressed murine MSCs *in vitro* with hypoxia, Lipopolysaccharide (LPS), and hydrogen peroxide and showed that female cells secreted more VEGF and less Tumor Necrosis Factor alpha (TNF-α) than male cells 16. A review by Ray *et al* showed that sex hormones influenced the characteristics of many types of mesenchymal stem cells, with effects that vary according to cell type 30.

There is no study about the effect of gender on MSCs efficacy in liver injury; however, some studies have investigated the efficacy of MSCs transplantation in other diseases. Deasy *et al* reported that the female muscle-derived stem cells could regenerate skeletal muscle more efficiently than male muscle-derived stem cells 31. In addition, in an experimental myocardial infarction mouse model, infusion of female-derived MSC resulted in more noticeable improvement in left ventricular function 32. On the other hand, there is some evidence that Haematopoietic Stem Cells (HSCs) in mice exhibited differences in cell-cycle regulation according to gender which results in more proliferation rate in female-derived HSC compared with male-derived HSC 33. The results of a study of 1,386 patients undergoing allogeneic HSC transplantation showed that sex matching between donors and recipients correlated with better overall survival, although HSCs from male donors were associated with better long-term survival 34. Jernberg *et al* showed that in pediatric leukemia, HSC transplantation from a female donor to a male recipient produced unfavorable outcomes comparing with all other sex combinations. They also demonstrated that the transplantation of cells derived from pregnant women to male patients increased the risk of graft-versus-host disease 35. Furthermore, in the experimental mice model of atherosclerosis, transplantation of Mononuclear Cells (MNCs) from the female bone marrow into male mice caused the release of anti-inflammatory and haematopoietic regulatory cytokines, whereas infusion of cells derived from the male bone marrow into a female recipient mouse did not demonstrate the same efficacy 36.

Researchers reported sex differences in cell behavior that may be of relevance in developing therapeutics; therefore, it was hypothesized that male recipients may not show noticeable improvement in liver function as well as female recipients. Surprisingly, our data showed that although MenSCs originated from females, but they can be equally effective in male recipients’ liver regeneration. It was shown that menstrual stem cells transplantation is not limited by sex.

## Conclusion

In summary, in this study, gender-specific MSCs were presented that can be used in human tissue engineering. Menstrual blood-derived stem cells could be collected from healthy women of reproductive age and banked for future use after expansion in culture. Engraftment of MenSCs in injured liver tissues of both genders of immunocompetent Balb/C mice caused a notable improvement in liver function assays. Our findings cleared the effect of recipient gender on MenSCs efficacy in acute liver injury. Further studies are needed to clarify the exact mechanism of sex differences in stem cells behaviors which results in improving the effectiveness of MenSCs transplantation.

## References

[1] Flores YN Lang CM Salmerón J Bastani R Risk factors for liver disease and associated knowledge and practices among Mexican adults in the US and Mexico. J Community Health 2012;37(2):403–411. 21877109 10.1007/s10900-011-9457-4

[2] Patton H Misel M Gish RG Acute liver failure in adults: an evidence-based management protocol for clinicians. Gastroenterol Hepatol (N Y) 2012;8(3):161–212. 22675278 PMC3365519

[3] Zarrinpar A Busuttil RW Liver transplantation: past, present and future. Nat Rev Gastroenterol Hepatol 2013; 10(7):434–440. 23752825 10.1038/nrgastro.2013.88

[4] Para O Crispino P Barone N Macis S Airasca L Gnerre P Sex differences in adverse drug reaction and liver disease. Italian J Med 2018;12(1):15–22.

[5] Liu WH Song FQ Ren Ln Guo WQ Wang T Feng YX The multiple functional roles of mesenchymal stem cells in participating in treating liver diseases. J Cell Mol Med 2015;19(3):511–520. 25534251 10.1111/jcmm.12482 PMC4369809

[6] Grompe M The role of bone marrow stem cells in liver regeneration. Semin Liver Dis 2003;23(4):363–372. 14722813 10.1055/s-2004-815560

[7] Yin L Zhu Y Yang J Ni Y Zhou Z Chen Y Adi-pose tissue-derived mesenchymal stem cells differentiated into hepatocyte-like cells in vivo and in vitro. Mol Med Rep 2015;11(3):1722–1732. 25395242 10.3892/mmr.2014.2935 PMC4270341

[8] Yun JW Ahn JH Kwon E Kim SH Kim H Jang JJ Human umbilical cord-derived mesenchymal stem cells in acute liver injury: Hepatoprotective efficacy, subchronic toxicity, tumorigenicity, and biodistribution. Regul Toxicol Pharmacol 2016;81:437–447. 27693706 10.1016/j.yrtph.2016.09.029

[9] Ullah I Subbarao RB Rho GJ Human mesenchymal stem cells-current trends and future prospective. Biosci Rep 2015;35(2):e00191. 25797907 10.1042/BSR20150025 PMC4413017

[10] Kazemnejad S Akhondi M-M Soleimani M Zarnani AH Khanmohammadi M Darzi S Characterization and chondrogenic differentiation of menstrual blood-derived stem cells on a nanofibrous scaffold. Int J Artif Organs 2012;35(1):55–66. 22307334 10.5301/ijao.5000019

[11] Khanjani S Khanmohammadi M Zarnani AH Talebi S Edalatkhah H Eghtesad S Efficient generation of functional hepatocyte-like cells from menstrual blood-derived stem cells. J Tissue Eng Regen Med 2015;9(11): E124–E134. 23505217 10.1002/term.1715

[12] Darzi S Werkmeister JA Deane JA Gargett CE Identification and characterization of human endometrial mesenchymal stem/stromal cells and their potential for cellular therapy. Stem Cells Transl Med 2016;5(9):1127–1132. 27245365 10.5966/sctm.2015-0190 PMC4996433

[13] Khanmohammadi M Khanjani S Edalatkhah H Zarnani A Heidari-Vala H Soleimani M Modified pro-tocol for improvement of differentiation potential of menstrual blood-derived stem cells into adipogenic line-age. Cell Prolif 2014;47(6):615–623. 25252214 10.1111/cpr.12133 PMC6496861

[14] Siegel G Kluba T Hermanutz-Klein U Bieback K Northoff H Schäfer R Phenotype, donor age and gender affect function of human bone marrow-derived mesenchymal stromal cells. BMC Medicine 2013;11(1):146. 23758701 10.1186/1741-7015-11-146 PMC3694028

[15] Heathman TR Rafiq QA Chan AK Coopman K Nienow AW Kara B Characterization of human mesenchymal stem cells from multiple donors and the implications for large scale bioprocess development. Biochemical Engineering J 2016;108:14–23.

[16] Crisostomo PR Markel TA Wang M Lahm T Lillemoe KD Meldrum DR In the adult mesenchymal stem cell population, source gender is a biologically relevant aspect of protective power. Surgery 2007;142(2):215–221. 17689688 10.1016/j.surg.2007.04.013

[17] Chen L Zhang C Chen L Wang X Xiang B Wu X Human menstrual blood-derived stem cells ameliorate liver fibrosis in mice by targeting hepatic stellate cells via paracrine mediators. Stem Cells Transl Med 2017; 6(1):272–284. 28170193 10.5966/sctm.2015-0265 PMC5442725

[18] Olsson RF Logan BR Chaudhury S Zhu X Akpek G Bolwell BJ Primary graft failure after myeloablative allogeneic hematopoietic cell transplant-ation for hematologic malignancies. Leukemia 2015;29 (8):1754–1762. 25772027 10.1038/leu.2015.75 PMC4527886

[19] Flowers ME Inamoto Y Carpenter PA Lee SJ Petersdorf EW Pereira SE Comparative analysis of risk factors for acute and for chronic graft-versus-host-disease according to National Institute of Health con-sensus criteria. Blood 2011 ;117(11):3214–3219. 21263156 10.1182/blood-2010-08-302109 PMC3062319

[20] Tajiri N Duncan K Borlongan MC Pabon M Acosta S de la Pena I Adult stem cell transplantation: is gender a factor in stemness? Int J Mol Sci 2014;15(9):15225–15243. 25170809 10.3390/ijms150915225 PMC4200754

[21] Fathi-Kazerooni M Tavoosidana G Taghizadeh-Jahed M Khanjani S Golshahi H Gargett CE Comparative restoration of acute liver failure by menstrual blood stem cells compared with bone marrow stem cells in mice model. Cytotherapy 2017;19(12):1474–1490. 29107739 10.1016/j.jcyt.2017.08.022

[22] Darzi S Zarnani AH Jeddi-Tehrani M Entezami K Mirzadegan E Akhondi MM Osteogenic differentiation of stem cells derived from menstrual blood versus bone marrow in the presence of human platelet releasate. Tissue Eng Part A 2012;18(15–16): 1720–1728. 22571521 10.1089/ten.tea.2011.0386 PMC3419854

[23] Berardis S Sattwika PD Najimi M Sokal E Use of mesenchymal stem cells to treat liver fibrosis: current situation and future prospects. World J Gastroenterol 2015;21(3):742–758. 25624709 10.3748/wjg.v21.i3.742 PMC4299328

[24] Di Bonzo LV Ferrero I Cravanzola C Mareschi K Rustichell D Novo E Human mesenchymal stem cells as a two-edged sword in hepatic regenerative medicine: engraftment and hepatocyte differentiation versus profibrogenic potential. Gut 2008;57(2):223–231. 17639088 10.1136/gut.2006.111617

[25] Ahmed SK Mohammed SA Khalaf G Fikry H Role of bone marrow mesenchymal stem cells in the treatment of CCL 4 induced liver fibrosis in albino rats: a histological and immunohistochemical study. Int J Stem Cells 2014; 7(2):87–97. 25473446 10.15283/ijsc.2014.7.2.87 PMC4249908

[26] Huang B Cheng X Wang H Huang W Wang D Zhang K Mesenchymal stem cells and their secreted molecules predominantly ameliorate fulminant hepatic failure and chronic liver fibrosis in mice respectively. J Transl Med 2016;14(1):1. 26861623 10.1186/s12967-016-0792-1 PMC4746907

[27] Yu F Ji S Su L Wan L Zhang S Dai C Adipose-derived mesenchymal stem cells inhibit activation of hep atic stellate cells in vitro and ameliorate rat liver fibrosis in vivo. J Formos Med Assoc 2015;114(2):130–138. 25678175 10.1016/j.jfma.2012.12.002

[28] Levine P McDaniel K Francis H Kennedy L Alpini G Meng F Molecular mechanisms of stem cell therapy in alcoholic liver disease. Dig Liver Dis 2014;46(5):391–397. 24440312 10.1016/j.dld.2013.11.015

[29] Tögel F Hu Z Weiss K Isaac J Lange C Westenfelder C Administered mesenchymal stem cells protect against ischemic acute renal failure through differentiation-independent mechanisms. Am J Physiol Renal Physiol 2005;289(1):F31–F42. 15713913 10.1152/ajprenal.00007.2005

[30] Ray R Novotny NM Crisostomo PR Lahm T Abarbanell A Meldrum DR Sex steroids and stem cell function. Mol Med 2008;14(7–8):493–501. 18475312 10.2119/2008-00004.Ray PMC2376641

[31] Deasy BM Schugar RC Huard J Sex differences in muscle-derived stem cells and skeletal muscle. Crit Rev Eukaryot Gene Expr 2008;18(2):173–188. 18304031 10.1615/critreveukargeneexpr.v18.i2.60

[32] Jiang W Ma A Wang T Han K Liu Y Zhang Y Intravenous transplantation of mesenchymal stem cells improves cardiac performance after acute myocardial ischemia in female rats. Transpl Int 2006;19(7):570–580. 16764636 10.1111/j.1432-2277.2006.00307.x

[33] Pietras EM Warr MR Passegué E Cell cycle regulation in hematopoietic stem cells. J Cell Biol 2011;195(5):709–720. 22123859 10.1083/jcb.201102131 PMC3257565

[34] Pond GR Lipton JH Messner HA Long-term survival after blood and marrow transplantation: comparison with an age-and gender-matched normative population. Biol Blood Marrow Transplant 2006;12(4):422–429. 16545726 10.1016/j.bbmt.2005.11.518

[35] Jernberg ÅG Remberger M Ringdén O Winiarski J Risk factors in pediatric stem cell transplantation for leukemia. Pediatr Transplant 2004;8(5):464–474. 15367282 10.1111/j.1399-3046.2004.00175.x

[36] Silvestre J-S Gojova A Brun V Potteaux S Esposito B Duriez M Transplantation of bone marrow–derived mononuclear cells in ischemic apolipoprotein E–knockout mice accelerates atherosclerosis without altering plaque composition. Circulation 2003;108(23):2839–2842. 14656923 10.1161/01.CIR.0000106161.43954.DF

